# Adaptor Identity Modulates Adaptation Effects in Familiar Face Identification and Their Neural Correlates

**DOI:** 10.1371/journal.pone.0070525

**Published:** 2013-08-21

**Authors:** Christian Walther, Stefan R. Schweinberger, Gyula Kovács

**Affiliations:** 1 DFG Research Unit Person Perception, Friedrich-Schiller-University of Jena, Jena, Germany; 2 Institute of Psychology, University of Regensburg, Regensburg, Germany; 3 Department for General Psychology and Cognitive Neuroscience, Friedrich-Schiller-University of Jena, Jena, Germany; 4 Institute of Psychology, Friedrich-Schiller-University of Jena, Jena, Germany; 5 Department of Cognitive Science, Budapest University of Technology and Economics, Budapest, Hungary; Tel Aviv University, Israel

## Abstract

Adaptation-related aftereffects (AEs) show how face perception can be altered by recent perceptual experiences. Along with contrastive behavioural biases, modulations of the early event-related potentials (ERPs) were typically reported on categorical levels. Nevertheless, the role of the adaptor stimulus per se for face identity-specific AEs is not completely understood and was therefore investigated in the present study. Participants were adapted to faces (S1s) varying systematically on a morphing continuum between pairs of famous identities (identities A and B), or to Fourier phase-randomized faces, and had to match the subsequently presented ambiguous faces (S2s; 50/50% identity A/B) to one of the respective original faces. We found that S1s identical with or near to the original identities led to strong contrastive biases with more identity B responses following A adaptation and vice versa. In addition, the closer S1s were to the 50/50% S2 on the morphing continuum, the smaller the magnitude of the AE was. The relation between S1s and AE was, however, not linear. Additionally, stronger AEs were accompanied by faster reaction times. Analyses of the simultaneously recorded ERPs revealed categorical adaptation effects starting at 100 ms post-stimulus onset, that were most pronounced at around 125–240 ms for occipito-temporal sites over both hemispheres. S1-specific amplitude modulations were found at around 300–400 ms. Response-specific analyses of ERPs showed reduced voltages starting at around 125 ms when the S1 biased perception in a contrastive way as compared to when it did not. Our results suggest that face identity AEs do not only depend on physical differences between S1 and S2, but also on perceptual factors, such as the ambiguity of S1. Furthermore, short-term plasticity of face identity processing might work in parallel to object-category processing, and is reflected in the first 400 ms of the ERP.

## Introduction

Human faces are a highly relevant stimulus class carrying manifold social signals, such as a person's identity, emotional state, gender, or age. Nevertheless, our perception of a given face is not necessarily identical at different points in time, as preceding visual experiences can influence it. The adaptation-related aftereffect (AE) is one such phenomenon, where prolonged exposure (i.e., adaptation) to a stimulus leads to contrastive biases in the perception of a subsequently presented stimulus. AEs have been described previously for lower level visual information such as line-orientation [1] or motion [2], but also for high-level information, including various characteristics of faces, among them a person's identity [3]–[6], gender [7]–[10], ethnicity, emotional expression [10], gaze [11], [12], and age [13].

In a recent study on face identity perception, Hills et al. [3] showed that adaptation to one familiar identity biases perception of a face morphed between this and another familiar identity towards the not adapted identity. Interestingly, the amount of AE depended on the type of the adaptor: adaptation to one of the faces that were used to create the morphs led to higher AEs than adaptation to different pictures of the same identities, while the strongest AEs were found for adaptation to artist drawn caricatures. Previous studies have also shown that AEs are influenced by adaptor and test stimulus presentation times [5], [14]. Longer adaptation and shorter test presentation increased the strength of AE. However, other studies suggest that AEs are relatively insensitive to variations of the adaptors, such as in contrast, colour, or size [15], viewpoint, inversion, or vertical stretching [6].

In all of these studies, AEs were typically induced by effective adaptors, such as the veridical or original faces of given identities, strong emotional expressions, or heavy distortions of faces. Some studies also found that adaptation to a face that is neutral on the adapted dimension does not lead to AEs, for example for facial distortions [16] or for facial identity [4]. Furthermore, “different image” adaptors (e.g., [3]), if included at all, were only used to rule out image-specific AEs. As of yet, a parametric study of the influence of the adaptor stimulus per se on the strength of AE has, to our knowledge, never been made.

Face AEs were often observed with modulations of event-related potentials (ERPs), and two groups of effects can be separated in the literature. Category-specific adaptation, especially of the N170 ERP component (or of its magnetic equivalent, the M170), was observed as reduced amplitudes for test faces following adaptation to faces as compared to stimuli of a different category [7], [8], [17]–[20]. Some studies also reported distortion-specific modulations of ERPs for test faces after adaptation to distorted faces [17], [21]. Compared to categorical adaptation effects on the N170, the time windows of those specific effects appeared more variable. Zimmer and Kovács [17] showed that N170 was additionally reduced after adaptation to distorted as compared to undistorted faces, whereas Burkhardt et al. [21] found the earliest effect after a similar manipulation in a later P250 component. Somehow at odds with findings of N170 being insensitive to the repetition of facial identity [19], [22], [23] or to familiarity per se [24], recent studies also reported a sensitivity of the N170 ERP to the identity of unfamiliar faces in an adaptation paradigm, but without addressing behavioural aftereffects (e.g., [25]).

A different line of experiments used functional magnetic resonance imaging (fMRI) to investigate the suppression of the blood oxygen level dependent (BOLD) signal in AE paradigms [26], [27]. Cziraki et al. [26] found face-specific response suppression in the fusiform face area (FFA; [28]) for composite face/hand stimuli after face adaptation, and this suppression was stronger in trials in which adaptation successfully biased perception away from the adaptor category. In an fMRI study on expression and identity AEs, Furl et al. [29] found similar response-specific effects in the medial temporal lobe, but not in the FFA or occipital face area (OFA; [30]). The functional mechanisms underlying such activation differences are still under discussion (for a review, see [31]). Furthermore, Rotshtein et al. [32] made an attempt to investigate categorical perception of face identity [33] without prior adaptation, to test the role of FFA and OFA in face recognition. They found that the inferior occipital gyrus showed sensitivity for the (physically driven) similarity of the stimuli, whereas the right fusiform gyrus responded to identity changes, implicating its role in identity recognition.

In a recent study on identity AEs [34], we asked how adaptation influences the perception of faces varying on morph continua between pairs of familiar identities. Clear AEs were only observed for ambiguous test stimuli if the adaptor was one of the original faces, while there was no AE following 50/50% morphs or Fourier phase randomized faces. Simultaneously recorded ERPs showed clear categorical adaptation for the N170, P2 and N250 components, as well as identity-specific adaptation effects for the P2 and N250, which were correlated with behavioural AEs. These results suggested an influence of ambiguity and/or (physical) similarity of the stimuli for AEs. However, as the design of Walther et al. [34] focused on relating AEs and priming to each other, it did not allow to draw specific conclusions about the role of adaptor similarity or ambiguity for AEs alone. Hence, in the present study, we investigated the stimulus-driven mechanisms behind the influence of the adaptor on the perception of the following test stimulus in an S1–S2 paradigm similar to Walther et al. [34]. Varying the identity of the adaptor (S1) parametrically on a morphing continuum, while keeping the test stimulus (S2) constant at an ambiguous level, we found a contrastive bias on the perception of S2 faces. The magnitude of AEs was systematically influenced by the S1 morph level, suggesting a relevance of both the physical differences between the adaptor and test stimuli, and perceptual factors, such as the ambiguity of the adaptors. Simultaneously recorded ERPs showed that S1 morph level affected brain responses in two relatively late time windows around 300–400 ms. In earlier time windows, especially around 125–240 ms, we observed categorical adaptation and response-specific effects.

## Materials and Methods

### Participants

Twenty-two right-handed students of the University of Jena (12 female) with a mean age of 23.3 years and a range of 20 to 27 years contributed data. All participants had normal or corrected to normal vision, gave written informed consent and received course credit for their participation. Data from five additional participants had to be excluded from the analysis due to technical problems (*N* = 3) or extensive EEG artifacts and drifts (*N* = 2). The experiment was carried out in accordance with the Declaration of Helsinki and was approved by the Ethics Committee of the Friedrich-Schiller-University of Jena.

### Stimuli

Stimuli comprised 28 famous faces (14 female) collected from the public domain of the world-wide web. We formed 14 same-gender pairs consisting of two unique identities (A and B) with as little associative relationship between the two members of the pairs as possible. For each of these 14 pairs, we created a morphing continuum between identities A and B (Sierra software Morph™, version 2.5) with eleven morph levels (corresponding to 10% steps, S1_100/0%_ to S1_0/100%_). These morphs, as well as Fourier phase randomized versions of faces (S1_N(oise)_) that were unique for each A–B pair, were used as first stimuli (S1s). S1_N_ served as a control stimulus matched on low level visual information and was created by MATLAB 7.6 (MathWorks Inc.) using an algorithm similar to Näsänen [35]. The second, target stimulus (S2) was always the ambiguous, 50/50% A/B morph of the identity pair that was presented as S1. Beforehand, images were prepared using Adobe Photoshop CS2 (Adobe Systems Inc.). Excessive hairstyles were cropped and faces were aligned to the same pupil position. Next, images were converted to greyscale and set to similar luminance and contrast values subjectively. Where appropriate, strands of hair, paraphernalia or extensive make-ups were manually removed. Final images measured 531×704 pixels. E-prime 2.0 software was used for stimulus presentation on a CRT monitor. S2 faces were presented at maximum visual angles of approximately 7.2×5.2 degrees, and S1 faces at 9.0×6.5 degrees, on a grey background. The mean luminance of the faces was 15 cd/m^2^ (measurement procedure as described in [34]). S1 stimuli were presented 25% larger than S2 faces to prevent effects based on retinal position.

### Procedure

Participants were seated in a dimly lit chamber 90 cm in front of the screen. A chin rest was used to reduce head movements during data recording to a minimum. Participants had to fixate the S1 stimulus, and to match the following S2 face to one of its original identities (A or B).

**Figure 1:**
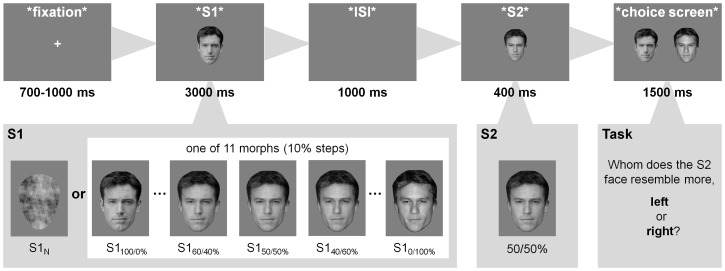
Trial structure of the experiment. Sample images belong to one of the 14 identity A/B pairs that were used in the experiment. Note that S1_50/50%_ and S2_50/50%_ are identical images, and that the expressions in asterisks are for illustration only and were not presented on the screen. The original images of identity A (Ben Affleck) and identity B (Heath Ledger) were obtained from http://wallpaper-s.org/72__Ben_Affleck,_Actor.htm (last access: 04/17/2013) and http://24.media.tumblr.com/tumblr_m1z1hcXtCM1qbps4ao1_500.jpg (last access: 04/17/2013), respectively.

Experimental trials (see Fig. 1) started with a variable fixation period of 700–1000 ms, after which the S1 was shown for 3000 ms. After an 1000 ms blank screen, the S2 was presented for 400 ms. Subsequently, participants had to match the morphed S2 face via button press (2-AFC) to one of its original identities (A or B, corresponding to S1_100/0%_ and S1_0/100%_), which were presented side by side on the choice screen for 1500 ms (positioned randomly, with identity A being presented on the left side for half of the trials, stimulus size: 7.2×5.2 degrees). A 10.8 degrees distance from image-centre to image-centre was used to prevent spatial overlap between the S2 and the faces of the choice-screen. Responses were recorded during the presentation of the choice screen, and the message “Bitte schneller reagieren!” (“Please respond faster!”) was displayed for 1000 ms if no response occurred in this time window.

The experiment consisted of two practice phases and an experimental phase. The experimental phase comprised 504 trials in three blocks (168 trials/block). Stimuli from all 14 A/B pairs were presented once per block in each of the 12 S1 conditions. Within blocks, stimulus order was random. The assignment of celebrities to labels A and B in each face pair was counterbalanced across participants. Participants were allowed to take a user-terminated rest every 56 trials. Trial procedure of the practice phases was similar to that of the experimental phase, except that different stimuli were presented that were not used in the experimental phase. Both practice phases comprised a representative portion of S1 conditions (i.e., S1_80/20%_, S1_50/50%_, S1_20/80%_, and S1_N_). In practice phase 1 (16 trials), unambiguous S2 stimuli and feedback were used to familiarize participants with the matching task, whereas stimulation in practice phase 2 (12 trials) was analogous to that in the experimental phase, i.e., 50/50% A/B morphs were used as S2, and no feedback was given. The total experimental time was about 62 min.

### Event-related Potential Recordings

The electroencephalogram (EEG) was recorded using a 64-channel Biosemi Active II system (Biosemi, Amsterdam, The Netherlands) at electrode positions Fp1, FT9, AF3, F1, F3, F5, F7, FT7, TP9, FC3, FC1, C1, C3, C5, T7, TP7, PO9, CP3, CP1, P1, P3, O9, P7, P9, PO7, PO3, O1, Iz, Oz, POz, Pz, CPz, Fpz, Fp2, FT10, AF4, Afz, Fz, F2, F4, F6, F8, FT8, TP10, FC4, FC2, FCz, Cz, C2, C4, C6, T8, TP8, PO10, CP4, CP2, P2, P4, O10, P8, P10, PO8, PO4 and O2 (according to the extended international 10/20 system). Note that the Biosemi system uses a combined ground/reference (CMS/DRL) circuit (cf. to http://www.biosemi.com/faq/cms&drl.htm). In addition, the horizontal electrooculogram (EOG) was recorded from the outer canthi of both eyes, and the vertical EOG bipolarly from above and below the left eye. The sampling-rate was 512 Hz (bandwidth: DC to 120 Hz). Offline we created 1200-ms-long epochs starting 200 ms before S2 onset. Ocular artifact correction was computed automatically with BESA 5.1.8.10 (MEGIS Software GmbH, Graefelfing, Germany) for all signals, and a recalculation to average reference was applied. Thresholds for artifact rejection were 100 µV for amplitude, 75 µV for gradient, and 0.1 µV for low signal. Trials with missing responses were excluded as well. On average, 39 trials per condition (i.e., ∼93% of all trials) were used for the analyses (range: 38–40 trials per condition). Finally, we calculated event related potentials (ERPs) by averaging the trials within each condition for each channel and participant. We additionally calculated ERPs depending on the response of the participants to S2 and the S1 identity (cf., e.g., [26]): for unambiguous S1s (corresponding to S1_100/0%_, S1_90/10%_, S1_80/20%_, S1_20/80%_, S1_10/90%_, and S1_0/100%_) we averaged all trials where the response was incongruent with the respective S1 identity (i.e., when adaptation biased perception away from the identity of the S1), as well as all trials with congruent responses (i.e., when adaptation did not lead to a contrastive bias of perception of the S2_50/50%_). Because there were about twice as many trials with successful compared to unsuccessful adaptation, we randomly included only every second trial with successful adaptation for this analysis, to achieve comparable trial numbers in both conditions (*M* = 76 trials/condition; range: 71 to 81 trials per condition). ERPs were digitally filtered with a 0.3 Hz high-pass (zero-phase shift, 6 dB/octave) and a 40 Hz low-pass filter (zero phase shift, 12 dB/octave). As we were interested in the role of S1 for the processing of S2 in the current study ERPs for S1 were not analysed.

### Behavioural Data Analysis

ANOVAs with repeated measures on S1 condition (11; S1_100/0%_ to S1_0/100%_ in 10% steps) were performed for S2 accuracies (in proportion endorsed as identity B) and S2 reaction times (RTs). Epsilon corrections for heterogeneity of covariance according to Huynh and Feldt (1976) were used throughout, where appropriate. Errors of omission (no key press) and trials with reaction times (RTs) faster than 200 ms were excluded from the analyses (in total, 0.7% of all experimental trials). To assess the character of the respective psychometric curves, we carried out polynomial contrast analyses with the ANOVAs. In addition, the difference between subsequent S1 morph levels, as well as between each face S1 condition and the chance level (0.5, i.e., 50%), were evaluated using *t*-tests (paired-samples and one-sample, respectively; two-sided). Paired-samples *t*-tests (two-sided) were used to determine if there was a significant difference between S1_50/50%_ and S1_N_ for accuracies and RTs. For accuracy data, S1_N_ was also compared to chance level using a one-sample *t*-test (two-sided). Post-hoc *t*-tests were not corrected for multiple comparisons. Only significant results or important trends are reported.

### ERP Data Analysis

We calculated mean amplitudes in the response to S2 for P1 (100–150 ms), N170 (125–175 ms), P2 (190–240 ms), and N250 (250–300 ms) time windows, as well as for two consecutive later time windows (300–350 ms and 350–400 ms) for each condition, electrode, and participant separately. With the exception of the two later time windows, for which no clear peaks were identifiable, time windows were centered on the respective peaks of the grand average across all conditions and participants at representative electrodes. In all following analyses, the topographic factors hemisphere (left vs. right) and electrode position (except for P1, which we only measured at O1 and O2) were included. N170 was quantified at P7/8, P9/10, PO7/8, and PO9/10. P2 and N250, as well as the two later time windows, were measured at P7/8, P9/10, PO7/8, PO9/10, O1/2, and O9/10. Note that the two later time windows are simply fixed 50-ms time segments that do not correspond to prominent peaks in the waveform, and thus any interpretation relative to known components should be considered with caution. For all ANOVAs, Epsilon corrections for heterogeneity of covariance according to Huynh and Feldt (1976) were used throughout, where appropriate. Post-hoc *t*-tests were not corrected for multiple comparisons. Topographic effects will only be reported when in interaction with the experimental variables.

To analyse *categorical adaptation effects*, we calculated the means across all face S1 conditions in every time window and compared these to the respective S1_N_ condition via ANOVAs with repeated measures on hemisphere, electrode, and S1 category (face vs. noise). Significant (or marginally significant) interactions of S1 category and topographic factors were tested post-hoc by comparing face S1s and noise S1s at each electrode separately (paired-samples *t*-tests, two-sided).

To assess *adaptation effects depending on S1 morph level*, repeated-measures ANOVAs including the factors hemisphere, electrode, and S1 condition (11; excluding S1_N_) were carried out for each component/time window. If the main ANOVA yielded an at least marginally significant interaction of S1 condition with a topographic factor, separate ANOVAs with repeated measures on S1 condition (11) and polynomial contrast analyses were carried out for each electrode of the respective overall analysis. If the effect of S1 condition was significant, we compared S1_100/0%_ and S1_0/100%_ to S1_50/50%_ with paired-samples *t*-tests (two-sided). To allow further comparisons to another recent study from our lab [34], we also tested the difference between S1_50/50%_ and the mean of S1_100/0%_ and S1_0/100%_ conditions in such cases in the same way.

For the analyses of *response-specific effects*, new mean amplitudes were calculated for the respective conditions. We used the same time windows and electrodes as given above, and ANOVAs with repeated measures on hemisphere, electrode position (except for P1), and response congruence (congruent vs. incongruent) were conducted. Significant (or marginally significant) interactions of response congruence and topographic factors were tested post-hoc with paired-samples *t*-tests (two-sided).

## Results

### Behavioural Data

**Figure 2:**
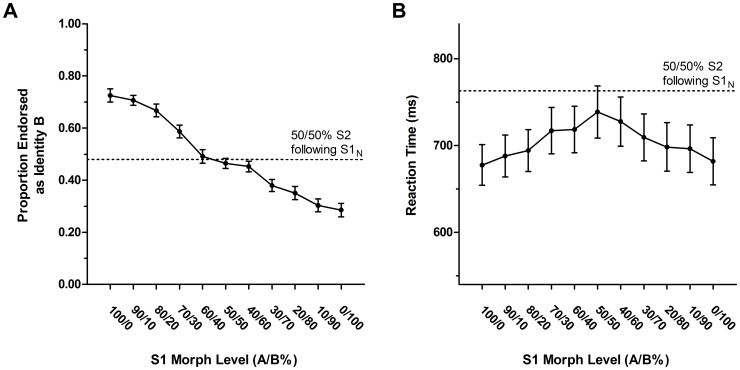
Behavioural data. A: Accuracy data (proportion endorsed as identity B) for S2_50/50%_ faces following the different S1 adaptors. B: Reaction times (in ms) for S2_50/50%_ faces following the different S1 adaptors. Error bars show ±1 standard error of the mean (SEM).

Varying the identity of S1 determined the participants' responses to S2 as suggested by the significant main effect of S1 condition in the ANOVA for classification data (*F*(10,210) = 48.88, *p*<.001, ε_HF_ = .220, η^2^_p_ = .699). The more similar the S1 was to one of the original identities, the stronger the classification of the ambiguous S2 face was biased towards the opposite identity and, therefore, the stronger was the contrastive aftereffect (see Fig. 2A). Polynomial contrast analyses revealed a strong linear trend (*F*(1,21) = 77.28, *p*<.001, η^2^_p_ = .786), but also significant quadratic (*F*(1,21) = 7.33, *p* = .013, η^2^_p_ = .259), fourth order (*F*(1,21) = 10.46, *p* = .004, η^2^_p_ = .333), and ninth order trends (*F*(1,21) = 5.23, *p* = .033, η^2^_p_ = .199), as well as marginally significant fifth and seventh order trends (*F*(1,21) = 4.17, *p* = .054, η^2^_p_ = .166, and *F*(1,21) = 3.29, *p* = .084, η^2^_p_ = .136, respectively). This suggests that the gradual change of physical difference between the ambiguous S2 and the different morph levels of S1 alone cannot explain the observed adaptation effect. The comparison of each S1 morph level with chance level confirmed that the previous presentation of the ambiguous S1_60/40%_ and S1_50/50%_ faces did not lead to perceptual biases (*p*s>.05), whereas all other S1 morph levels led to a classification performance significantly different from chance (*p*s<.05). The comparison of S2s following subsequent morph levels (see Table 1) showed differences between all subsequent pairs except for S1s near the unambiguous, as well as the most ambiguous S1 morph levels. Therefore, perception of the ambiguous S2 seems to be influenced by certain S1 morph levels in much the same way, indicating a step-wise shape of the curve.

**Table 1:** t-tests of differences in the proportion of S2 being endorsed as identity B between subsequent S1 morph levels.

Difference	*M*	*SD*	*t* (21)	*p*
S1_100/0%_ – S1_90/10%_	0.02	0.09	1.00	.328
S1_90/10%_ – S1_80/20%_	0.04	0.09	2.10	**.048**
S1_80/20%_ – S1_70/30%_	0.08	0.12	3.26	**.004**
S1_70/30%_ – S1_60/40%_	0.10	0.08	5.28	**.000**
S1_60/40%_ – S1_50/50%_	0.03	0.12	0.99	.332
S1_50/50%_ – S1_40/60%_	0.01	0.12	0.50	.623
S1_40/60%_ – S1_30/70%_	0.07	0.07	5.05	**.000**
S1_30/70%_ – S1_20/80%_	0.03	0.06	2.42	**.025**
S1_20/80%_ – S1_10/90%_	0.05	0.06	3.58	**.002**
S1_10/90%_ – S1_0/100%_	0.02	0.07	1.20	.244

*Note:* Significant *p*-values are in boldface.

The ANOVA for S2 RTs showed a main effect of S1 condition (*F*(10,210) = 10.82, *p*<.001, ε_HF_ = .885, η^2^_p_ = .340), that was best described by a strong quadratic trend (*F*(1,21) = 41.63, *p*<.001, η^2^_p_ = .665; see Fig. 2B). This suggests that the closer the S1 morph was to one of the original identities, the faster the responses to the ambiguous S2 were. The polynomial contrast analyses revealed also fourth order (*F*(1,21) = 12.40, *p* = .002, η^2^_p_ = .371), and sixth order trends (*F*(1,21) = 5.78, *p* = .026, η^2^_p_ = .216), as well as marginally significant ninth and tenth order trends (*F*(1,21) = 4.05, *p* = .057, η^2^_p_ = .162, and *F*(1,21) = 3.20, *p* = .088, η^2^_p_ = .132, respectively).

The S1_N_ condition, that was introduced as an additional control, was neither significantly different from chance (*t*(21) = −1.30, *p = *.207), nor from the S1_50/50%_ condition, (*t*(21) = −0.85, *p = *.403), for S2 classification data. Nevertheless, there was a marginally significant difference between S1_N_ and S1_50/50%_ for S2 RTs, suggesting that the slowest responses were observed following S1_N_ (*t*(21) = −1.83, *p = *.082).

### ERP Data

#### Categorical adaptation effects

**Figure 3:**
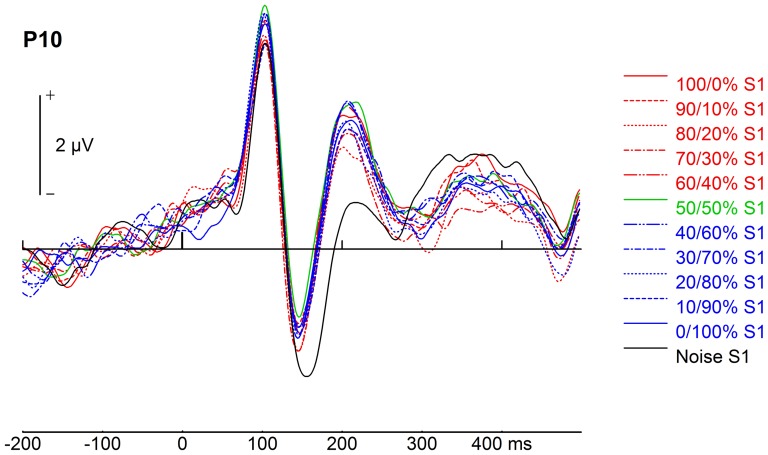
Sample ERPs. ERPs for 50/50% S2 faces following the eleven S1 morphs at P10 electrode, plotted from −200 to 500 ms. Note that the choice screen onset was at 400 ms.

**Figure 4:**
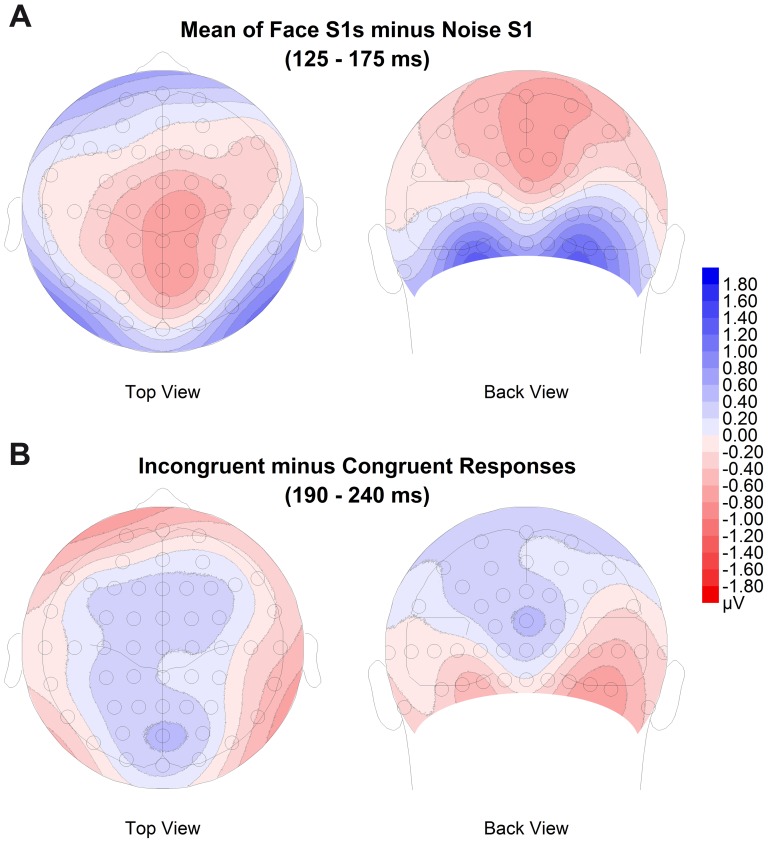
Topographical difference maps. A: ERPs to S2 preceded by face S1s minus those preceded by the Noise S1 (S1_N_; 125–175 ms). B: Trials with incongruent minus congruent responses (190–240 ms). All maps were created using spherical spline interpolation and show a 110 degrees equidistant projection from a top and back view perspective (including electrode positions).

To assess categorical adaptation, i.e. adaptation related to generic face exposure [7], we averaged the S2 ERPs of the eleven S1 conditions where S1 was a face, and then contrasted this average with the S1_N_ condition (see also the difference between all face S1s and S1_N_ in Fig. 3). The first component reflecting categorical adaptation was the P1, quantified by a main effect of S1 category (*F*(1,21) = 13.46, *p* = .001, η^2^_p_ = .391). Irrespective of hemisphere, the mean amplitudes of P1 were larger for S2s following face S1s as compared to S1_N_. Importantly, the most prominent categorical adaptation effects were observed for the N170 and the P2 ERP components. Both components showed significant main effects of S1 category (*F*(1,21) = 15.11, *p* = .001, η^2^_p_ = .418, and *F*(1,21) = 29.20, *p*<.001, η^2^_p_ = .582, for N170 and P2, respectively), which were qualified by interactions of electrode and S1 category (*F*(3,63) = 10.08, *p*<.001, ε_HF_ = .738, η^2^_p_ = .324, and *F*(5,105) = 7.05, *p* = .002, ε_HF_ = .434, η^2^_p_ = .251, for N170 and P2, respectively). Corresponding to prior findings (e.g., [17], see their Figures 3A and 3B), adaptation to face S1s led to smaller N170 amplitudes and larger P2 amplitudes than adaptation to S1_N_ over most of the occipito-temporal electrodes. Post-hoc tests for N170 revealed significant differences between S2s following face S1s and S1_N_ at P9, PO9, P8, P10, and PO10 (*p*s<.05), as well as PO8 (*t*(21) = 1.73, *p = *.099). These differences were significant at all analysed electrodes for P2 (*p*s<.05), except for O2 (*t*(21) = 1.54, *p = *.138). N250 also showed categorical adaptation reflected by an interaction of electrode and S1 category (*F*(5,105) = 7.53, *p*<.001, ε_HF_ = .609, η^2^_p_ = .264). N250 mean amplitudes were significantly larger (i.e., less positive) for S2s following face S1s as compared to S1_N_ at P7, PO7, and O1 (*p*s<.05), as well as O2 (*t*(21) = −1.76, *p* = .093), suggesting somewhat more pronounced effects over superior occipito-temporal sites. Finally, we also found significantly smaller (i.e., less positive) mean amplitudes for S2s following face S1s as compared to S1_N_ irrespective of electrode in the analyses of the two later time windows, as shown by main effects of S1 category in the 300–350 ms and 350–400 ms time windows (*F*(1,21) = 13.71, *p* = .001, η^2^_p_ = .395, and *F*(1,21) = 8.15, *p* = .009, η^2^_p_ = .280, respectively). Topographical difference maps for the 125–175 ms time window (corresponding to the N170 ERP component; see Fig. 4A) suggested that categorical adaptation was most pronounced over occipito-temporal regions of both hemispheres, confirming the results of mean amplitudes analyses.

#### Adaptation effects depending on S1 morph level

**Figure 5:**
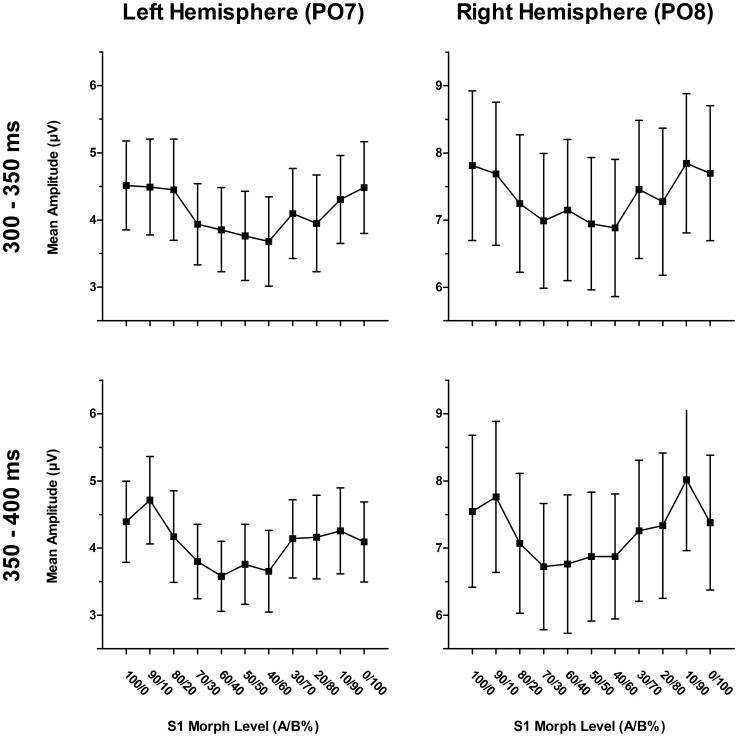
ERP effect of S1 condition. Mean amplitudes (300–350 ms and 350–400 ms) for 50/50% S2 faces following the eleven S1 morphs for one representative electrode pair (PO7 and PO8). Error bars show ±1 standard error of the mean (SEM).

We found no effect of S1 condition for P1, N170, and N250 ERP components. Although there was a marginally significant interaction of electrode and S1 condition in the analyses of P2 (*F*(50,1050) = 1.52, *p* = .063, ε_HF_ = .432, η^2^_p_ = .068), its pattern was rather unsystematic. When tested separately only the P10 electrode showed an effect of S1 condition (*F*(10,210) = 2.03, *p* = .033, η^2^_p_ = .088), which was fitted best by cubic and fourth order trends (*F*(1,21) = 5.02, *p* = .036, η^2^_p_ = .193, and *F*(1,21) = 10.06, *p* = .005, η^2^_p_ = .324, respectively). In contrast to the effects observed for the earlier components, there were prominent modulations of S2 ERPs by S1 condition in both the 300–350 ms and 350–400 ms time windows at occipito-temporal sites (see Fig. 5 and Figs. S1, S2, S3), as shown by significant main effects of S1 condition (*F*(10,210) = 2.33, *p* = .013, η^2^_p_ = .100, and *F*(10,210) = 4.20, *p*<.001, η^2^_p_ = .167, respectively). These effects were further qualified by a significant interaction of electrode and S1 condition in the 300–350 ms time window (*F*(50,1050) = 1.79, *p* = .010, ε_HF_ = .510, η^2^_p_ = .079), and a marginally significant interaction of electrode and S1 condition in the 350–400 ms time window (*F*(50,1050) = 1.50, *p* = .062, ε_HF_ = .474, η^2^_p_ = .067). However, the effects of S1 condition were qualitatively similar in both time windows and observable at several electrodes, as revealed by post-hoc ANOVAs (see Tables 2 and 3). In general, the closer the S1 face was to one of the original faces, the larger (less negative) the S2 mean amplitudes were. This pattern was confirmed by strong quadratic trends in the data, but sometimes modified by additional linear or higher order trends (see Tables 2 and 3). Furthermore, post-hoc *t*-tests showed significant differences between S1_100/0%_ and S1_50/50%_ at PO7, PO9, O1, and PO8 (*p*s<.05) in the 300–350 ms time window, and significant differences at PO7, PO9, and O1 (*p*s<.05), as well as a trend at O2 (*t*(21) = 1.91, *p = *.070), in the 350–400 ms time window. S1_0/100%_ and S1_50/50%_ differed significantly at PO7, O1, and PO8 (*p*s<.05) in the 300–350 ms time window, while in the 350–400 ms time window, there were only trends for such a difference at O1 and O2 (*t*(21) = 1.83, *p = *.081, and *t*(21) = 1.72, *p = *.099, respectively). The difference between S1_50/50%_ and the mean of S1_100/0%_ and S1_0/100%_ was significant at PO7, O1, and PO8 (*p*s<.05) in the 300–350 ms time window, while in the 350–400 ms time window, this difference was significant at PO7 (*t*(21) = −2.34, *p = *.029) and O1 (*t*(21) = −2.48, *p = *.022), and marginally significant at O2 (*t*(21) = −2.02, *p = *.056). Note that the latter difference seems to rely too much on a symmetric modulation of ERPs by S1 condition and is therefore not optimal in the context of the present experiment. Together with the topographical effects, revealed by the main ANOVAs, the post-hoc results suggest that S1 morph level dependent adaptation effects are somewhat less pronounced over more temporal electrodes, especially in the 300–350 ms time window.

**Table 2:** Post-hoc ANOVA with repeated measures on S1 condition (11) at each of the occipito-temporal electrodes in the 300–350 ms time window.

Electrode	Main Effect of S1 Condition	Polynomial Contrasts
P7	*F*(10,210) = 1.97, *p* = .053, ε_HF_ = .795, η^2^_p_ = .086	Quadratic: *F*(1,21) = 7.66, *p* = .012, η^2^_p_ = .267
PO7	*F*(10,210) = 2.37, *p* = .011, η^2^_p_ = .102	Quadratic: *F*(1,21) = 27.21, *p*<.001, η^2^_p_ = .564
PO9	*F*(10,210) = 2.02, *p* = .032, η^2^_p_ = .088	Quadratic: *F*(1,21) = 7.63, *p* = .012, η^2^_p_ = .267
O1	*F*(10,210) = 3.06, *p* = .001, η^2^_p_ = .127	Quadratic: *F*(1,21) = 21.56, *p*<.001, η^2^_p_ = .507
P8	*F*(10,210) = 2.23, *p* = .029, ε_HF_ = .778, η^2^_p_ = .096	Quadratic: *F*(1,21) = 5.84, *p* = .025, η^2^_p_ = .218
		4th Order: *F*(1,21) = 5.54, *p* = .028, η^2^_p_ = .209
PO8	*F*(10,210) = 2.73, *p* = .004, η^2^_p_ = .115	Quadratic: *F*(1,21) = 16.67, *p*<.001, η^2^_p_ = .443

*Note:* The analysed electrodes were P7, P9, PO7, PO9, O1, O9, P8, P10, PO8, PO10, O2, and O10. Only (marginally) significant S1 condition effects and significant polynomial trends are reported.

**Table 3:** Post-hoc ANOVA with repeated measures on S1 condition (11) at each of the occipito-temporal electrodes in the 350–400 ms time window.

Electrode	Main Effect of S1 Condition	Polynomial Contrasts
P7	*F*(10,210) = 2.38, *p* = .011, η^2^_p_ = .102	Linear: *F*(1,21) = 5.03, *p* = .036, η^2^_p_ = .193
PO7	*F*(10,210) = 3.13, *p* = .003, ε_HF_ = .803, η^2^_p_ = .130	Quadratic: *F*(1,21) = 28.14, *p*<.001, η^2^_p_ = .573
		4th Order: *F*(1,21) = 9.99, *p* = .005, η^2^_p_ = .322
PO9	*F*(10,210) = 3.42, *p*<.001, η^2^_p_ = .140	Quadratic: *F*(1,21) = 12.57, *p* = .002, η^2^_p_ = .369
		Cubic: *F*(1,21) = 5.15, *p* = .034, η^2^_p_ = .197
O1	*F*(10,210) = 2.73, *p* = .004, η^2^_p_ = .115	Quadratic: *F*(1,21) = 13.57, *p* = .001, η^2^_p_ = .393
O9	*F*(10,210) = 3.16, *p* = .002, ε_HF_ = .838, η^2^_p_ = .131	Quadratic: *F*(1,21) = 7.25, *p* = .014, η^2^_p_ = .257
		Cubic: *F*(1,21) = 4.44, *p* = .047, η^2^_p_ = .175
		4th Order: *F*(1,21) = 9.03, *p* = .007, η^2^_p_ = .301
P8	*F*(10,210) = 2.53, *p* = .007, η^2^_p_ = .107	Quadratic: *F*(1,21) = 7.24, *p* = .014, η^2^_p_ = .256
		4th Order: *F*(1,21) = 5.34, *p* = .031, η^2^_p_ = .203
PO8	*F*(10,210) = 3.39, *p* = .001, ε_HF_ = .796, η^2^_p_ = .139	Quadratic: *F*(1,21) = 10.60, *p* = .004, η^2^_p_ = .335
		4th Order: *F*(1,21) = 4.45, *p* = .047, η^2^_p_ = .175
		6th Order: *F*(1,21) = 9.88, *p* = .005, η^2^_p_ = .320
PO10	*F*(10,210) = 2.42, *p* = .009, η^2^_p_ = .103	Quadratic: *F*(1,21) = 14.40, *p* = .001, η^2^_p_ = .407
		6th Order: *F*(1,21) = 4.84, *p* = .039, η^2^_p_ = .187
O2	*F*(10,210) = 3.40, *p*<.001, η^2^_p_ = .139	Quadratic: *F*(1,21) = 11.86, *p* = .002, η^2^_p_ = .361
		4th Order: *F*(1,21) = 7.35, *p* = .013, η^2^_p_ = .259
		6th order: *F*(1,21) = 6.35, *p* = .020, η^2^_p_ = .232
O10	*F*(10,210) = 2.19, *p* = .019, η^2^_p_ = .095	Quadratic: *F*(1,21) = 11.46, *p* = .003, η^2^_p_ = .353
		4th Order: *F*(1,21) = 4.59, *p* = .044, η^2^_p_ = .179

*Note:* The analysed electrodes were P7, P9, PO7, PO9, O1, O9, P8, P10, PO8, PO10, O2, and O10. Only (marginally) significant S1 condition effects and significant polynomial trends are reported.

#### Response-specific effects

**Figure 6:**
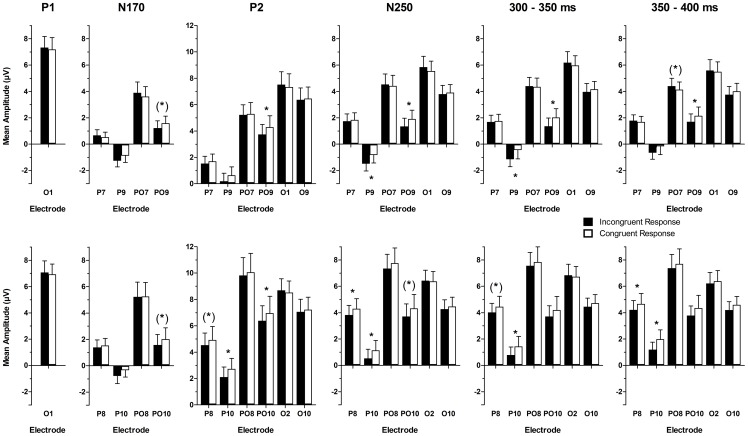
Response-specific effects. Mean amplitudes of trials with incongruent (i.e., adaptation biased perception of S2 away from the identity of the S1) and congruent responses (i.e., adaptation did not lead to a contrastive bias of perception of the S2_50/50%_) for all analysed components and electrodes over left (top row) and right hemisphere (bottom row). Significant differences between incongruent and congruent responses and trends are marked: (*) – *p*<.10; * – *p*<.05. Error bars show ±1 standard error of the mean (SEM).

We found first hints for effects of the congruence of S1 and the behavioural response to the S2 (see Materials and Methods section) in the N170 time window (see Fig. 6). N170 mean amplitudes were larger when perception was biased away from the S1, i.e., the response was incongruent to the S1, as described by a significant interaction of electrode and response congruence (*F*(3,63) = 4.33, *p* = .029, ε_HF_ = .525, η^2^_p_ = .171). Post-hoc tests showed trends at PO9 and PO10 (*t*(21) = −1.97, *p* = .062, and *t*(21) = −1.76, *p* = .094, respectively). For P2, mean amplitudes were significantly smaller (i.e., relatively more negative) for incongruent responses, i.e., when adaptation biased perception away from the identity of the adaptor, as indicated by an interaction of electrode and response congruence (*F*(5,105) = 6.02, *p* = .001, ε_HF_ = .660, η^2^_p_ = .223). Post-hoc tests showed significant differences between conditions with congruent and incongruent responses at PO9, P10, and PO10 (*p*s<.05), as well as a trend at P8 (*t*(21) = −1.97, *p* = .062), indicating slightly larger effects over the right hemisphere. Although there was no interaction of hemisphere and response congruence in the overall ANOVA, a slight right lateralization of the occipito-temporal effects was also suggested by topographical difference maps for the P2 time window (see Fig. 4B). Response-specific effects were also observed in the N250 time window, again as a significant interaction of electrode and response congruence (*F*(5,105) = 7.03, *p*<.001, ε_HF_ = .786, η^2^_p_ = .251). The N250 mean amplitudes were larger (i.e., less positive/more negative) for S2 faces following incongruent responses as compared to congruent responses at P9, PO9, P8, and P10 (*p*s<.05), as well as at PO10 (*t*(21) = −2.01, *p* = .058). In the two later time windows, mean amplitudes were lower (i.e., less positive/more negative) for S2 faces following incongruent when compared to congruent responses. This was quantified by a significant interaction of electrode and response congruence (*F*(5,105) = 4.91, *p* = .007, ε_HF_ = .508, η^2^_p_ = .189) in the 300–350 ms time window, and significant interactions of hemisphere and response congruence, as well as electrode and response congruence in the 350–400 ms time window (*F*(1,21) = 5.31, *p* = .032, η^2^_p_ = .202, and *F*(5,105) = 3.24, *p* = .038, ε_HF_ = .494, η^2^_p_ = .133, respectively). Post-hoc tests revealed that the amplitude reduction for incongruent responses was significant at P9, PO9, and P10 (*p*s<.05), and marginally significant at P8 (*t*(21) = −1.81, *p* = .085) in the 300–350 ms time window, as well as at PO9, P8, and P10 (*p*s<.05) in the 350–400 ms time window, with an additional, but opposed trend at PO7 (*t*(21) = 1.88, *p* = .074).

## Discussion

In the present study, we investigated the effect of parametric manipulation of S1 identity by morphing on AEs in familiar face perception. We replicated the findings of typical adaptation studies in showing contrastive biases in the perception of ambiguous faces after adaptation to veridical S1 faces [3], [6], as well as the lack of such effects following adaptation to ambiguous S1 morphs [4], [10], [34]. Additionally, the strength of AEs was influenced by the S1 morph level systematically, in that AEs were smaller for S1s closer to the most ambiguous morph and stronger for S1s closer to the original faces. The observed influence of S1 morph level suggests that in addition to the physical differences between S1 and S2, perceptual factors, such as stimulus ambiguity, affect face identity AEs as well. This conclusion is supported by the comparison of accuracy differences between S1 pairs with 10% physical dissimilarity on our morphing continuum as well.

In analogy to studies of other face AEs, the observed effects of the current study might involve high-level representations of face identities, that were adapted selectively depending on the perceived identity of the S1 [6]. This idea is linked to studies showing that face identity is perceived categorically [32], [33]. Although we could not test for categorical perception explicitly in our design (the target face was always the same 50/50% ambiguous face), the non-linear contributions to the effect of S1 morph level suggest that categorical perception of the S1 might indeed be involved in AEs. AEs were stronger for S1s lying away from the boundary between two identities, while virtually no AE was observed for S1s close to the category boundary. Note that, although the linear trend in the effect of S1 morph level suggests the involvement of lower-level processes in the observed AEs as well, a merely retinotopic locus of AEs was ruled out by introducing a size change from S1 to S2 in the present study (see, e.g., [36]). Although separating high- and low-level contributions to face AEs is rather difficult, an account combining categorical perception with opposite AEs [37] could be helpful to further investigate the involvement of physical similarity and high-level identity information in face identity AEs.

RTs were also modulated by the identity of S1, in that participants responded faster following S1s near or equal to one of the original identities as compared to ambiguous S1s. Larger biases in the perception of target faces were therefore associated with faster RTs, possibly because the percept after effective adaptation was less ambiguous and easier to classify. Such an effect can be seen in extension to studies describing the benefits on discrimination performance associated with face adaptation [38], [39]. Furthermore, a recent computational modeling study of adaptation-related aftereffects [40] also suggested that a higher amount of adaptation leads to faster RTs for face stimuli. Additionally, we found a marginal RT benefit of face adaptation as compared to noise adaptation, but no image-specific priming effect for 50/50% morphs, that was described earlier in a similar gender discrimination study by Kaiser et al. (unpublished data).

In the present study, we found categorical adaptation effects in ERPs which are broadly consistent with those reported in other studies [8], [17], [19], [34]. In detail, ERPs over occipito-temporal recording sites were different if S1 was a face or a noise stimulus and this effect was most pronounced in the N170 and P2 time windows. However, a degree of categorical adaptation was already seen in the P1 component. Although some studies suggested that object category might be processed in such an early time window (e.g., [41]), this is controversial [42] and a number of recent studies state that face category processing does not occur before the N170 time window (e.g., [18], [19]). In comparison to the earlier effects, the N250 effects of the present study were rather weak and seemed limited to the more superior electrodes over the left hemisphere, whereas categorical adaptation was more pronounced in both the 300–350 ms and 350–400 ms time windows. In a recent study on the neural correlates of face AEs and priming [34], we observed similar effects of N170, P2, and N250, but the design of that study did not allow us to analyse later time windows. As studies on categorical adaptation typically focused on earlier ERP components (e.g., [8], [19]) as well, our study is the first to show that later S2 ERPs can be modulated by the category of S1. However, further research is needed to validate this finding. Also note that in our previous study [34], we observed categorical adaptation on the N250 as less positive/more negative mean amplitudes for S2s following noise S1s over more inferior occipito-temporal electrodes, whereas in the present study, more positive/less negative amplitudes for S2s following S1_N_ were observed over the more superior occipito-temporal recording sites. The differences between the present categorical adaptation effects on P1 and N250 and those reported earlier [34] might be due to different carry-over adaptation effects between trials, possibly introduced by the randomization of face and noise S1 trials. Altogether, the categorical effects observed in the present study suggest that the processing of stimulus category takes place in a relatively long time interval, encompassing the early ERP components, such as P1 and N170, as well as the P2, N250, and the time between 300 and 400 ms post stimulus.

In ERPs, our analyses also revealed effects of the manipulation of S1 identity (i.e., S1 morph level) in two relatively late time windows (300–350 ms and 350–400 ms) at occipito-temporal sites. Here, S2 mean amplitudes were more positive following unambiguous S1s as compared to ambiguous S1s. As the relationship between S1 morph level and amplitude is not linear, but rather quadratic, the signals did not seem to be modulated by the mere physical differences of S1 and S2, but might also reflect the influence of perceptual factors, such as the ambiguity of the S1. This might be related to the activation of an identity-specific area in right fusiform gyrus that was described by Rotshtein et al. [32]. Nevertheless, future research will have to confirm this finding and its interpretation, that has to remain somewhat speculative at present. We did not observe any systematic effects of S1 identity manipulation for earlier ERPs on the S2, such as the N250r, that is thought to be the first component reflecting individual face recognition [23], [43]. A possible reason for this might be that the N250r effects of the previous studies were typically measured as an increased negativity for familiar faces following images of the same as compared to a different identity, whereas in the present study the ambiguous S2 test face was not a familiar or identifiable face. Although some studies reported N170 modulations by facial identity (e.g., [25]), early components such as P1 and N170 were often described as insensitive to short term repetition priming for familiar face identities [19], [23], [44] and face familiarity per se [24]. Nevertheless, one might ask why the manipulation of S1 morph level, that involved a gradual physical change from one S1 image to another, failed to modulate pictorial encoding or structural encoding stages of face processing, as reflected by P1 and N170, respectively (for a review, see [45]). However, our S1 stimuli were equalized in lower-level properties such as relative size, luminance, and contrast (see Materials and Methods section), and therefore, the differences introduced by morphing may have been too subtle to affect these processes selectively. Finally, the present non-existent or unsystematic early ERP effects of S1 condition could reflect a power problem related to the moderate number of trials. That said, the systematic and significant late ERP effects of S1 condition between 300 and 400 ms would seem to argue against such a general power problem.

With the large number of unambiguous S1s used, we were also able to analyse trials where participants' response to S2 was incongruent to the S1 (i.e., adaptation successfully biased perception away from the adaptor identity) as compared to trials where perception was congruent to the S1 (i.e., adaptation did not lead to repelling aftereffects). Besides effects on N170, N250, and later time-windows, there was a prominent reduction of P2 mean amplitudes when adaptation lead to contrastive AEs as compared to when it did not. This effect could be related to studies showing increased suppression of the fMRI signal in trials where adaptation biased perception of an ambiguous test face away from the adaptor [26], [29], and suggests that amplitude modulations by adaptation to a specific face identity are related to perceptual decisions about ambiguous faces as measured with behaviour. The P2 is also thought to reflect task difficulty [46]. However, a novel study by Banko et al. [47] showed that it rather reflects increased perceptual processing demands, related to the presence of stimulus noise. In the present study, the decrease of P2 amplitude was specific to the response given while stimulation and task were identical and might therefore also reflect a lower difficulty of the decision when adaptation led to contrastive AEs as compared to when it did not. The results of other recent studies [48]–[50] suggested that a decrease in P2 amplitude is associated with decreasing typicality, or increased distinctiveness, of a test face in reference to a face space [51]. Note that the present results of the P2 are perfectly consistent with those recent findings, if one assumes that adaptation elicits a transient shift of the centre of face space towards the adaptor (e.g., [16]) in those trials in which adaptation successfully elicited contrastive AEs. We hold that in those trials (compared to trials where adaptation did not lead to a contrastive bias), the very same ambiguous S2 likely has been perceived as more distinctive and characteristic of the respective celebrity. Accordingly, those recent effects of caricaturing on the P2 and the present reduction of the P2 component during successful adaptation trials may reflect highly related phenomena in high-level face perception.

As qualitative differences between the processing of familiar and unfamiliar faces were sometimes suggested [52], it is unclear whether the effects observed in the present study and other studies on face identity AEs (e.g., [3], [6]) would generalize to unfamiliar face perception. For example, faces which are more readily recognized might lead to stronger maximal AEs via stronger activations of high-level representations of the individual faces in the face processing network, possibly reflected by modulations of the later ERP time windows. A recent study by Laurence and Hole [53] reported no difference in face distortion AEs between famous and unfamiliar faces, while reduced AEs were found only for the own face of the participants, suggesting only a moderate influence of familiarity on such configural AEs. Additionally, we observed clear behavioural AEs using a very similar paradigm to that of the present study with experimentally familiarized faces (unpublished data), although during this familiarization, faces were only shown from one perspective for several seconds. As, to our knowledge, there is no data on the role of familiarity for face identity AEs as of yet, such that further studies are needed to clarify this issue.

In conclusion, our results revealed that face identity AEs are modulated (1) by parametric manipulation of S1 identity, and also (2) not just by physical differences, but also by perceptual factors, such as the ambiguity of S1 adaptors. Furthermore, our data suggest a benefit of adaptation to unambiguous S1s for response speed. ERPs revealed systematic correlates of short-term plasticity of face identity processing within the first 400 ms following S2 onset. Finally, we also observed categorical adaptation during this time window, suggesting that the processing of stimulus category and identity may partially overlap in time.

## Supporting Information

Figure S1 **ERP effect of S1 condition on the P1 and N170 components.** Mean amplitudes for 50/50% S2 faces following the eleven S1 morphs at all analysed electrodes for P1 (100–150 ms) and N170 (125–175 ms) time windows. Error bars show ±1 standard error of the mean (SEM).
(TIF)

Figure S2 **ERP effect of S1 condition on the P2 and N250 components.** Mean amplitudes for 50/50% S2 faces following the eleven S1 morphs at all analysed electrodes for P2 (190–240 ms) and N250 (250–300 ms) time windows. Error bars show ±1 standard error of the mean (SEM).
(TIF)

Figure S3 **ERP effect of S1 condition in the late time windows.** Mean amplitudes for 50/50% S2 faces following the eleven S1 morphs at all analysed electrodes for the 300–350 ms and 350–400 ms time windows. Error bars show ±1 standard error of the mean (SEM).
(TIF)
