# Supplementary figures and images for: Adaptor Identity Modulates Adaptation Effects in Familiar Face Identification and Their Neural Correlates
Source: PLoS One. 2013 Aug 21;8(8):e70525. doi: 10.1371/journal.pone.0070525 (PMC3749171; doi:10.1371/journal.pone.0070525)

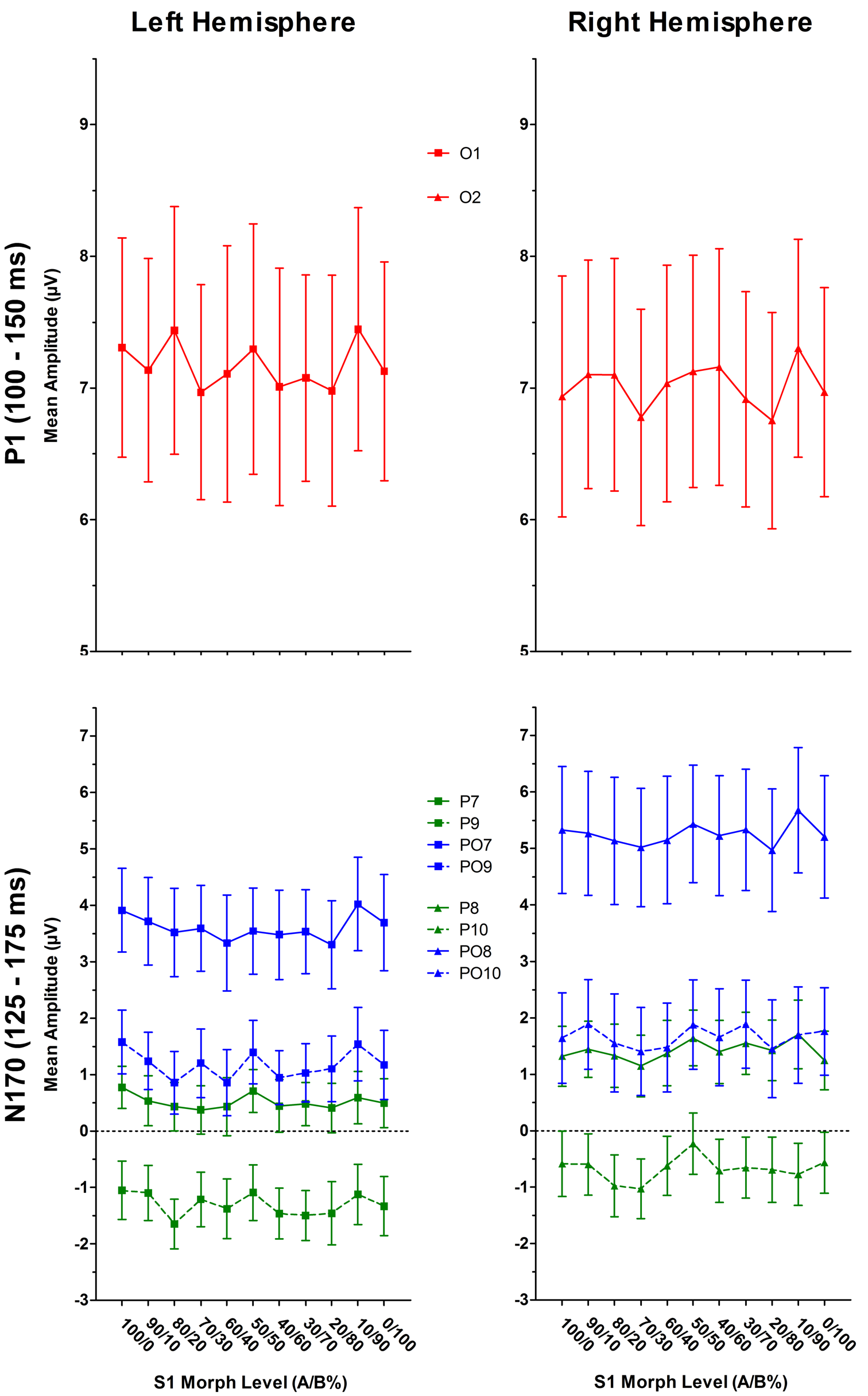

Supplement: Figure S1 — ERP effect of S1 condition on the P1 and N170 components. Mean amplitudes for 50/50% S2 faces following the eleven S1 morphs at all analysed electrodes for P1 (100–150 ms) and N170 (125–175 ms) time windows. Error bars show ±1 standard error of the mean (SEM). (TIF) [file pone.0070525.s001.tif]

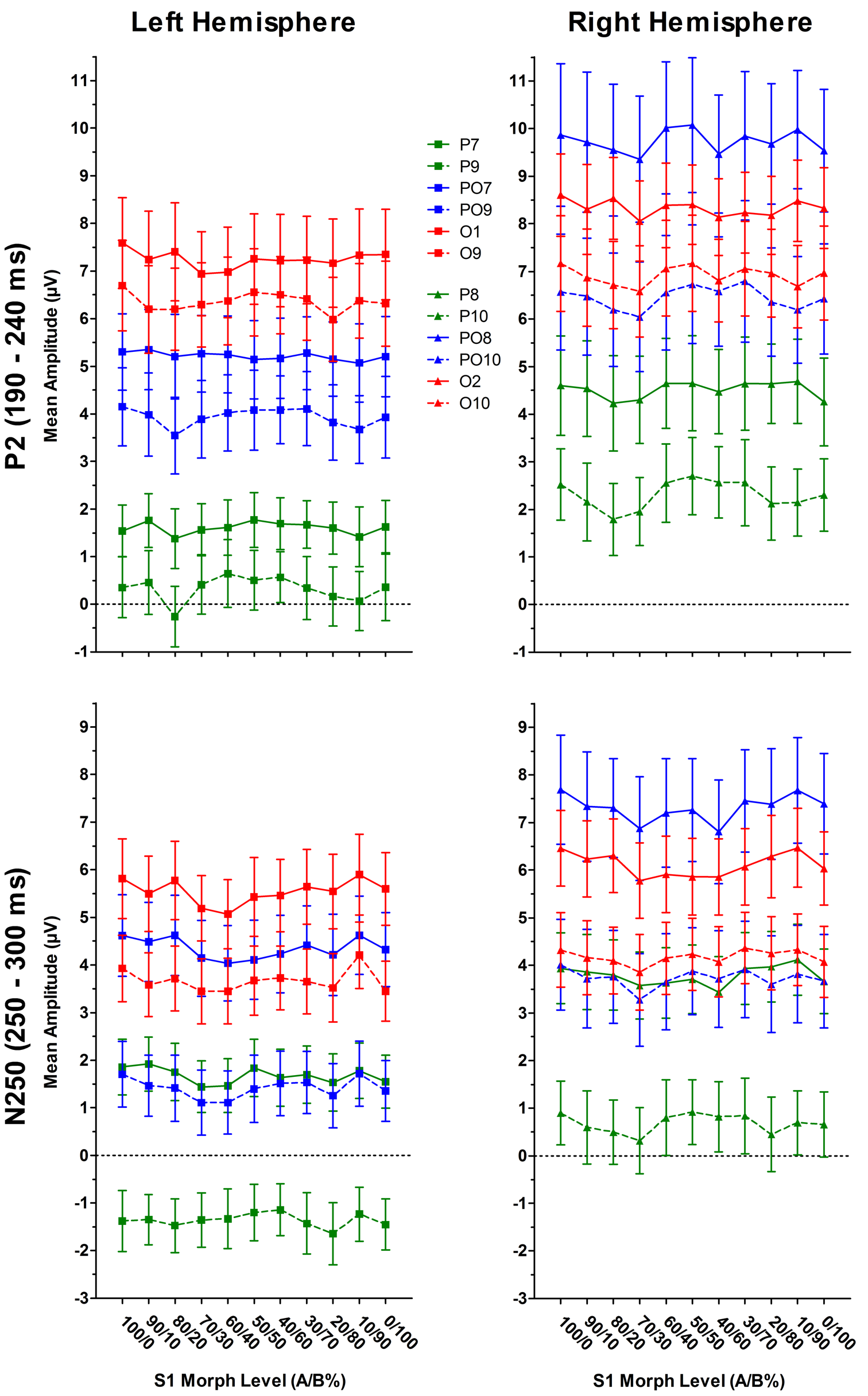

Supplement: Figure S2 — ERP effect of S1 condition on the P2 and N250 components. Mean amplitudes for 50/50% S2 faces following the eleven S1 morphs at all analysed electrodes for P2 (190–240 ms) and N250 (250–300 ms) time windows. Error bars show ±1 standard error of the mean (SEM). (TIF) [file pone.0070525.s002.tif]

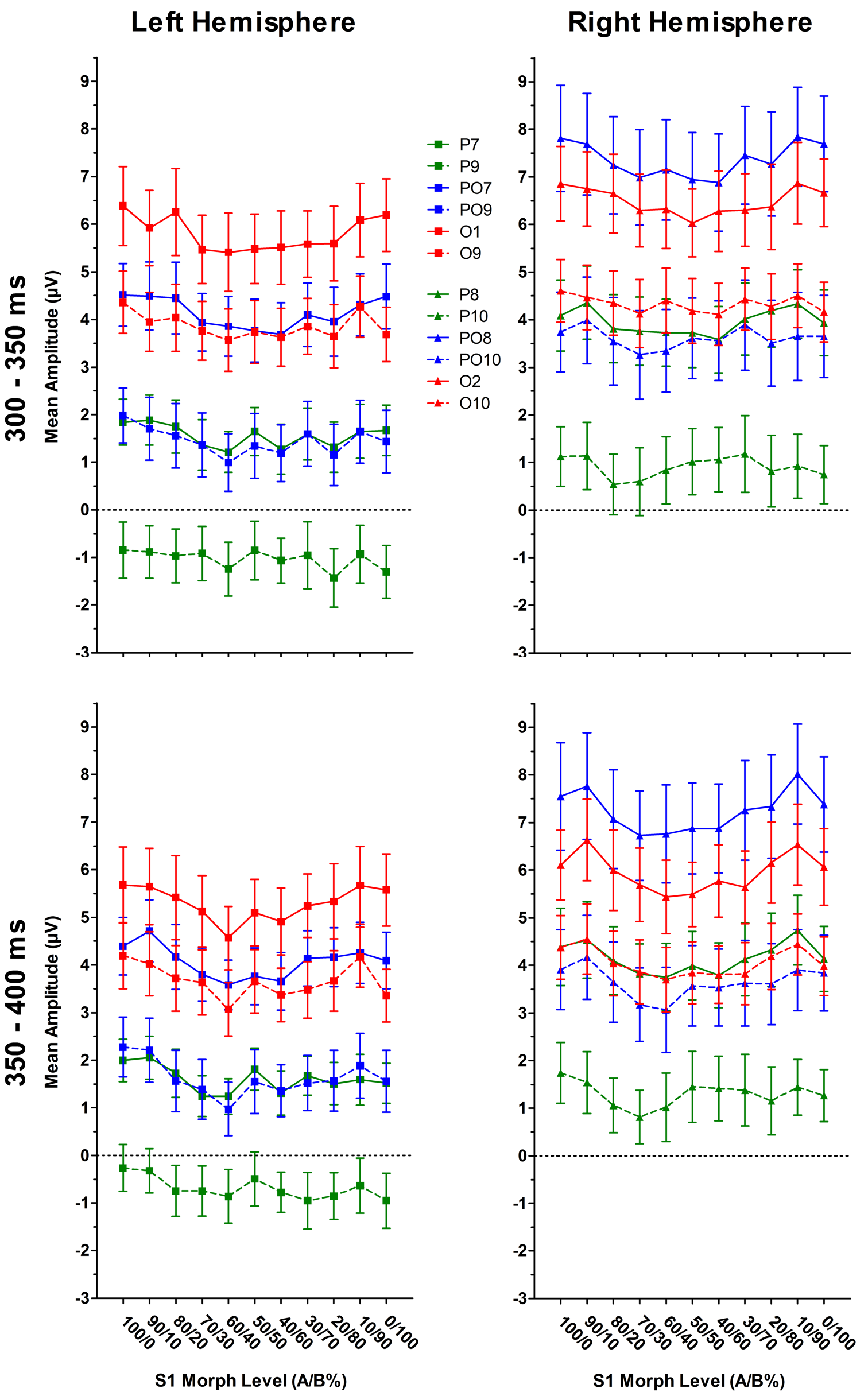

Supplement: Figure S3 — ERP effect of S1 condition in the late time windows. Mean amplitudes for 50/50% S2 faces following the eleven S1 morphs at all analysed electrodes for the 300–350 ms and 350–400 ms time windows. Error bars show ±1 standard error of the mean (SEM). (TIF) [file pone.0070525.s003.tif]
